# Recent Advances in Photoluminescence Detection of Fingerprints

**DOI:** 10.1100/tsw.2001.76

**Published:** 2001-10-02

**Authors:** E. Roland Menzel

**Affiliations:** Center for Forensic Studies, Texas Tech University, Lubbock, TX 79409, USA

## Abstract

Photoluminescence detection of latent fingerprints has over the last quarter century brought about a new level of fingerprint detection sensitivity. The current state of the art is briefly reviewed to set the stage for upcoming new fingerprint processing strategies. These are designed for suppression of background fluorescence from articles holding latent prints, an often serious problem. The suppression of the background involves time-resolved imaging, which is dealt with from the perspective of instrumentation as well as the design of fingerprint treatment strategies. These focus on lanthanide chelates, nanocrystals, and nanocomposites functionalized to label fingerprints.

### Contents

Introduction
Photoluminescence Detection of Fingerprints: Current Practice
Time-resolved Photoluminescence Imaging: Instrumentation
- Gated Imaging
- Phase-resolved Imaging

Time-resolved Photoluminescence Imaging: Fingerprint Treatments
- Lanthanide-based Fingerprint Development
- Fingerprint Labeling Strategies
- Lanthanide-based Fingerprint Development Revisited

Quantum Dots and Nanoparticles
- CdS/Dendrimer Nanocomposites
- Instrumentation Adaptation

## INTRODUCTION

When a finger touches a surface, on the order of 0.1 mg of residue is left behind, comprising a latent fingerprint. Typically, 98–99% of this is water, which soon evaporates to leave behind on the order of a microgram of material, which is made up of, in roughly equal quantities, inorganic salts and a complex mixture of organic constituents, including fatty acids, triglycerides, amino acids, vitamins, squalene, urea, etc. The visualization of latent fingerprints mostly involves targeting a class of these organic components. The early methods for fingerprint visualization involved dusting, iodine fuming, and silver nitrate. The first two of these involve preferential adherence to the fingerprint residue by physical processes, whereas the silver nitrate treatment is a chemical one that targets chloride anions in the latent fingerprint. This latter method is an analog of silver halide photography. The three methods date back to the early 1900s. In the early 1960s, the ninhydrin chemical fingerprint treatment began to see wide use. Ninhydrin attacks amino acids of fingerprint residue to form a purple product. These main methods, and some others of more specialized use, have long been the stuff of textbooks[1,2,3]. The visualization of fingerprints via these procedures is colorimetric, based on absorp-tion/reflection principles. In 1976, photoluminescence began to be explored as a generally applicable methodology to fingerprint detection[4]. The rationale for fingerprint detection based on photoluminescence is as follows: Let us consider a fingerprint on a white surface dusted with a black powder. Its visualization involves the discrimination between the intensity of ambient light reflected (typically diffusely) from locations between fingerprint ridges, and from the fingerprint ridges themselves. When the fingerprint is strong, no reflection is obtained from ridge locations because there the black powder absorbs all the incident light. When the fingerprint is weak, however, as mostly encountered in practice, only a little powder adheres to latent fingerprint ridges. They now reflect as well, slightly less intensely than the locations between ridges. The fingerprint visualization thus amounts to the detection of a small difference between two relatively large light signals. This is quite generally an insensitive detection mode. Provided that the background fluorescence from the article holding the latent fingerprint is not appreciable, a matter to be taken up shortly, the visualization of a fingerprint via photoluminescence involves light emitted only from the fingerprint ridges. The visualization of a weak fingerprint thus amounts to the detection of a small signal, rather than a small difference between large signals, and this can be done with high sensitivity. Given today's detection technology, which approaches single-photon sensitivity, the photoluminescence approach, if implemented correctly, should be ideal.

## PHOTOLUMINESCENCE DETECTION OF FINGERPRINTS: CURRENT PRACTICE

It was the intent in the initial exploration in 1976 of photoluminescence detection of fingerprints to devise a nondestructive, noninvasive technique such that, in case of failure, all other procedures could still be applied subsequently. The approach involved the excitation by light of fluorescence in constituents that naturally are present in fingerprint residue. The excitation must match the absorption of such constituents in order to produce fluorescence. The detection simply involved the visual observation (in a darkened room to eliminate ambient light) of the luminescent fingerprint through a filter that blocked the excitation light reflected from the article under scrutiny, but that transmitted the fingerprint luminescence. Subsequently, the fingerprint would be photographed through the same filter. Since very small quantities of material, of picomole order, are targeted in the fingerprint residue, a high-intensity excitation source is required to produce a luminescence visible to the naked eye, with luminescence intensity proportional to excitation intensity. Thus, high-power lasers became the excitation sources of choice for maximum sensitivity. Fingerprint residue absorbs primarily in the deep ultraviolet, at wavelengths shorter than 300 nm, not surprisingly, given the abundance of small organic molecules in the residue. This is not a generally useful wavelength domain because the resulting photoluminescence would typically occur in the near ultraviolet, not visible to the eye. Moreover, there is a dearth of user-friendly lasers for the deep UV. Fortunately, however, there are fluorescent components in fingerprint residue that absorb in the blue-green (and emit in the yellow-green). At the time, powerful and user-friendly lasers operating in this range were on hand, namely argon-ion lasers (of powers in the range of 5–20 W). Filtered lamps were also investigated, but, because their useful powers were only a few hundred mW, they sacrificed sensitivity. Inherent fingerprint fluorescence detection was found to work, and soon became successful in criminal casework[5,6]. The Valerian Trifa Case[6] is of particular note. It involved the detection of a 41-year-old fingerprint. It became clear very early on that all too many articles were not amenable to examination for inherent fingerprint fluorescence because of very intense background fluorescence.

There are two remedies to the background fluorescence problem that immediately come to mind, namely to devise fingerprint treatments that either increase the fingerprint luminescence intensity or that render it of a color different from that of the background in order to permit optical filtering for background suppression. Because one encounters in casework a great variety of articles and fingerprints of a range of ages, one must have on hand a corresponding range of fingerprint treatments. For instance, dusting is applicable only to relatively fresh fingerprints on smooth surfaces. Porous items, such as paper, call for chemical procedures instead. By 1979, a number of such treatments had been devised, including dusting with fluorescent powder and staining with fluorescent dye (both for smooth surfaces), along with several chemical treatments[5]. With the advent in the early 1980s of cyanoacrylate fuming[7], which can be followed very effectively by subsequent staining with fluorescent dyes, the dye staining approach has become very effective indeed. When ninhydrin (**I** of Fig. 1) reacts with the amino acids of fingerprint residue, a purple product, often referred to as Ruhemann's purple (**II** of Fig. 1) is formed. It was found in the early 1980s that subsequent treatment of this product with zinc chloride produces a highly fluorescent complex (**III** of Fig. 1) that responds to blue-green excitation[8].

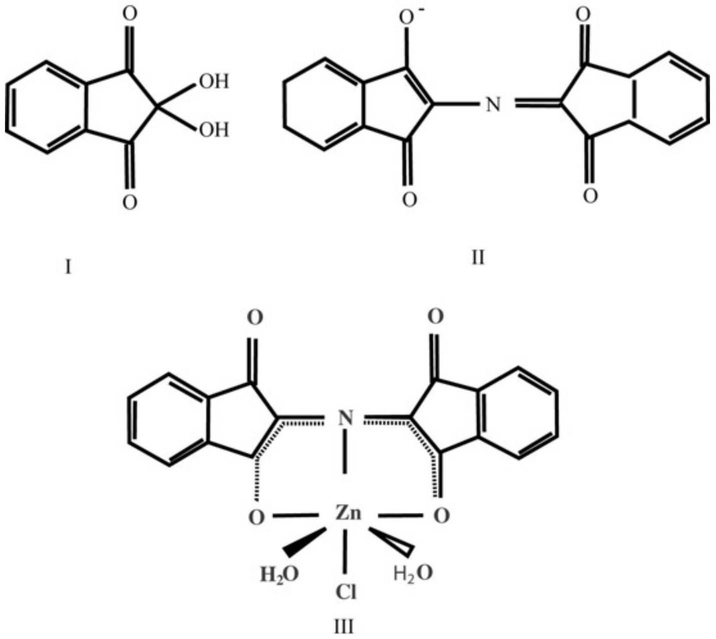

**FIGURE 1.**

This procedure has become routine for fingerprint detection on paper. It has the special virtue of being compatible with the traditional ninhydrin development, which remains in wide use today. The subsequent zinc chloride treatment and photoluminescence detection greatly increase fingerprint detectability. Since the early 1980s, a variety of additional physical and chemical fingerprint treatments for photoluminescence detection have been developed, including ninhydrin analogs and diazafluorenone, for instance. They, too, have by now become textbook material[9,10]. A recent Japanese article[11] reports that increased fluorescence intensity is obtained when zinc chloride is replaced by indium chloride, following ninhydrin or ninhydrin analogs (see [9] and [10] for comprehensive lists of references). The argon-ion laser is today still the most widely used laser for fingerprint work. Because of portability (ability to do crime scene examination instead of transporting evidence to the crime laboratory) and price, filtered lamps are today widely used also, but sacrifice sensitivity. The recent advent of powerful diode-pumped, frequency-doubled Nd:$YVO_4$ and similar lasers, which provide powers in the 5–10 W range at 532 nm, and which are portable, will, no doubt, advance current practice. Photography for the recording of photoluminescent fingerprints is becoming replaced by digital cameras. Thus, direct input into Automated Fingerprint Identification Systems (AFIS) and image processing for improved fingerprint detail extraction are now at hand. Given that the current state of photoluminescence detection of fingerprints is extensively documented, this mini-review will not belabor it further, but will focus, instead, on upcoming fingerprint treatments designed for background fluorescence suppression in concert with time-resolved imaging.

## TIME-RESOLVED PHOTOLUMINESCENCE IMAGING: INSTRUMENTATION[9,10,13]

Notwithstanding the success of the current procedures for photoluminescence detection of fingerprints, many kinds of articles (wood, for instance) remain recalcitrant because of very intense background fluorescence which, in addition, is spectrally broad, so that optical filtering to suppress it is not feasible. More sophisticated techniques of background suppression are thus called for in order to permit successful scrutiny.

### Gated Imaging

When the excitation light source is suddenly turned off, the photoluminescence decays exponentially. The lifetime of this decay is, in the case of typical offending background fluorescences, on the order of a nanosecond. If one were to devise a fingerprint treatment that leads to a luminescence lifetime substantially longer than this, one could then turn on the imaging device after light source cut-off, with a delay such that the background fluorescence has already decayed whereas substantial fingerprint luminescence remains. The procedure could be done repetitively, as depicted in Fig. 2. The scheme is referred to as gated imaging. In the fingerprint context, its feasibility was first explored and demonstrated in 1979[12], utilizing a rotating cylinder with two slots, as shown in Fig. 3. These served to turn the excitation light source on and off and also provided the gate width of the imaging device, an ordinary photographic camera. The position of the camera defined the gate delay. The fingerprint treatment involved dusting with a powder containing a terbium complex which luminesces with a lifetime on the order of a millisecond. While the device and the dusting powder served to demonstrate feasibility, the device was of no practical casework value for two reasons: First, one is limited in the speed of rotation of the cylinder, which limits one to fingerprint treatments that yield very long lifetimes.

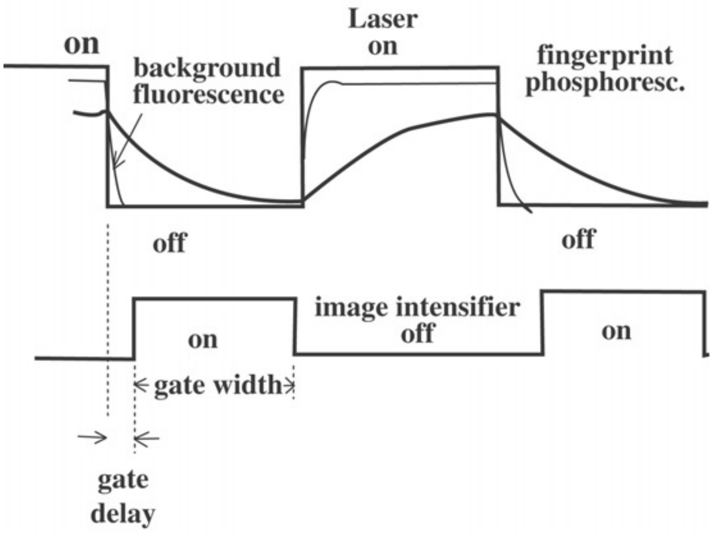

**FIGURE 2.**

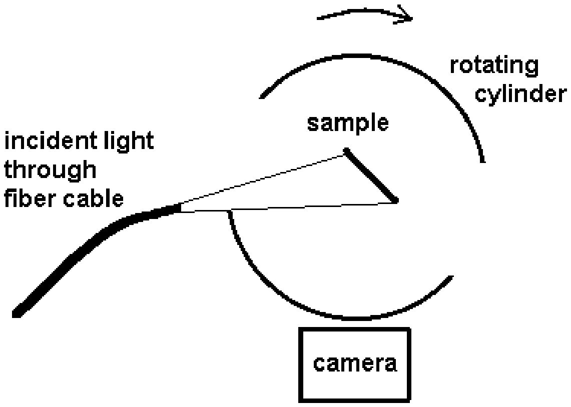

**FIGURE 3.**

Second, one cannot arbitrarily scale up the size of the cylinder, into which the sample has to be placed, if for no other reason than that eventually the distance between camera and sample becomes too large to retain adequate image resolution. The situation changed in the mid-1980s with the advent of proximity-focused microchannel plate image intensifiers (MCP). These can be electronically turned on and off very rapidly, in times on the order of $10^{-8}$ seconds, and they can serve as the front end of computer-interfaced CCD or other digital cameras. Fig. 4 shows the block diagram of an instrument that has been operational in the author's laboratory since 1992[14]. The system is suitable for casework, regardless of the size of the article to be examined. The light modulator shown in Fig. 4 may be an ordinary mechanical light chopper (a rotating wheel with holes in it), when millisecond lifetimes pertain, or it may be an electro-optic modulator, for fingerprint luminescence lifetimes down to about $10^{-7}$ seconds. In addition to "full frame" imaging, as depicted in Fig. 4, scanning gated imaging has also been implemented[15]. The scanning system is slow, but has the virtues of operating with a low-power laser and a light detector that is inexpensive compared to an intensified gateable CCD camera. When lifetimes become shorter than about $10^{-7}$ seconds, complete laser intensity cut-off becomes difficult to achieve, and different time-resolved imaging approaches are called for.

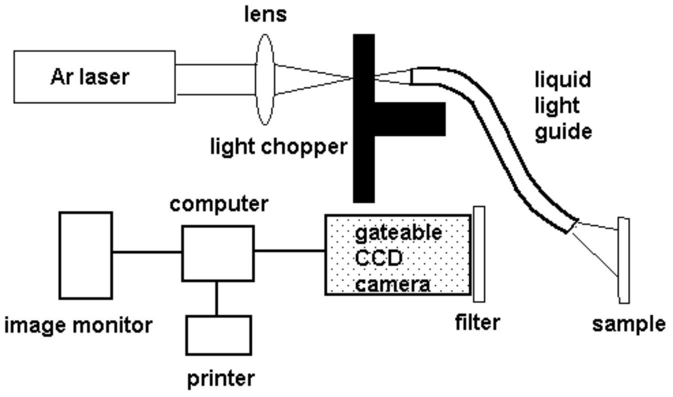

FIGURE 4.

## Phase-resolved Imaging

Let us suppose that the intensity of a luminescence excitation light source is modulated sinusoidally with modulation angular frequency $\omega$, namely $2\pi f$, where f is the modulation frequency. The resulting luminescence will then also be sinusoidal in intensity, but it will be phase delayed with respect to the excitation by a phase ($\phi$) that is related to the luminescence lifetime ($\tau$), as shown in Fig. 5. There also will be a lifetime-related demodulation of the luminescence (m), namely a variation in the sinusoidal luminescence intensity fluctuation, as also shown in Fig. 5. In this figure, the excitation and luminescence intensities are normalized to each other. The relationships between phase delay and demodulation and modulation angular frequency is shown in Fig. 6. Because ultimately the phase relationship between excitation and luminescence is the basis of the imaging for purposes of background fluorescence suppression, it is not necessary that the excitation intensity modulation be complete (i.e., zero at the lowest intensity level). Thus, very high modulation frequencies can be achieved, such that luminescence lifetimes in the nanosecond, and even picosecond, domain become accessible. The discrimination between a background luminescence and a sample luminescence requires only that their luminescence lifetimes be substantially different. In principle, the sample luminescence could have a lifetime shorter than that of the background, unlike the gated case, in which it must be

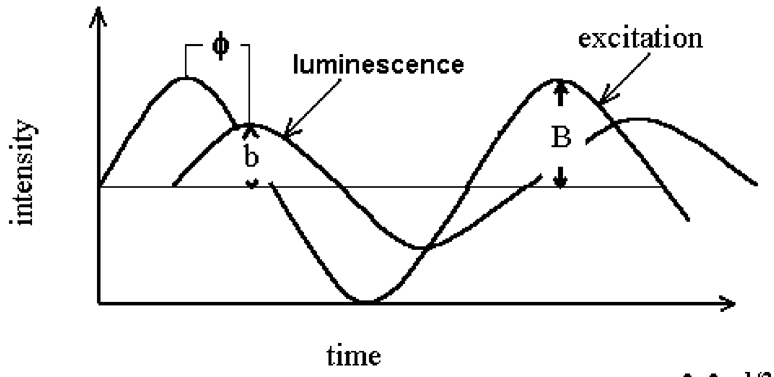

$\tan \phi = \omega\tau$ $\qquad m = b/B = (1 + \omega^2\tau^2)^{-1/2}$

FIGURE 5.

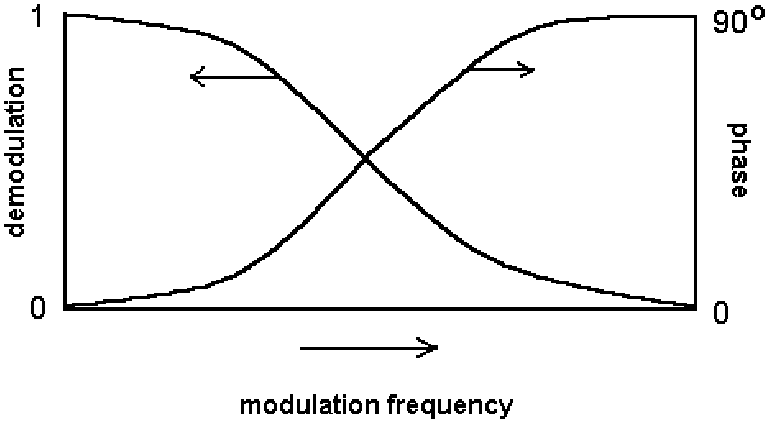

**FIGURE 6.**

longer than that of the background. In practice, however, one strives for long sample luminescence lifetimes because short ones, less than nanoseconds, are generally accompanied by low luminescence quantum efficiencies. For fingerprint detection, phase-resolved imaging systems have yet to be constructed, but they have been in operation for some time already in cell microscopy, for instance. There, such systems contain an added complexity, namely high-resolution microscopy. A basic version of a phase-resolved imaging system for fingerprint work is shown in Fig. 7. More sophisticated potential versions have been described in the literature (see Menzel[16] and references therein). The technology for time-resolved fingerprint imaging can be considered essentially mature. The crux of its implementation is the treatment to which fingerprints have to be subjected in order to yield the requisite long-lived luminescence (as compared to the background).

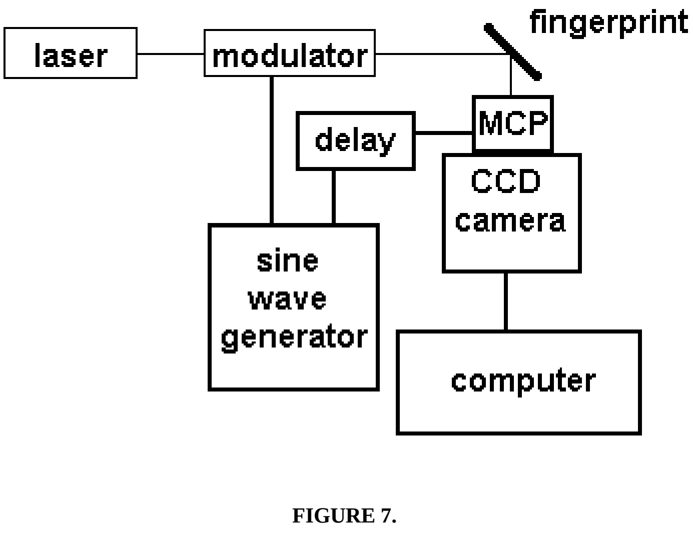

**FIGURE 7.**

## TIME-RESOLVED PHOTOLUMINESCENCE IMAGING: FINGERPRINT TREATMENTS

For purposes of devising fingerprint treatment strategies, it is convenient to divide surfaces holding latent fingerprints into two general types, smooth and porous. The former can be dealt with by dusting or staining; the latter type calls for chemical processing. A time-resolved strategy, in order to be of universal value, must be applicable to both types of article. Three general strategies are currently under study; all of them can be effectively applied to smooth surfaces. Feasibility has also been demonstrated for chemical development on porous surfaces, but chemical recipes have not yet reached the maturity for routine practical implementation. Accordingly, the focus below will be on chemical development of fingerprints, where maturity, although not realized yet, is in the offing. Two properties must be present in such chemical development: First, the reagent must preferentially attack a component of the fingerprint residue. Second, the reagent must have spectroscopic properties that lend themselves to time-resolved imaging. The reagent may serve both purposes simultaneously, or a mixed ligand complex may be used, in which one ligand serves the labeling function and the other the spectroscopy function. As an aside, it is to be recognized that any practical fingerprint detection chemistry requires commercial availability of reagents.

### Lanthanide-based Fingerprint Development[10]

Certain lanthanides, most notoriously $Eu^{3+}$ and $Tb^{3+}$, emit (red and green, respectively) with lifetimes of millisecond order. They are thus in principle well-suited for facile gated imaging. Most studies to date have focused on $Eu^{3+}$, which emits in the red under ultraviolet excitation. This large discrepancy between excitation and emission has the virtue that in many instances background fluorescence can be suppressed by optical filtering alone. The most common europium compound is $EuCl_3 \cdot 6H_2O$. One immediately notes that, accordingly, $Eu^{3+}$ prefers to exist in ninefold coordination. When the above salt is dissolved in a clear solvent, one obtains an essentially colorless solution. This indicates that $Eu^{3+}$ absorbs rather poorly, hence will emit poorly. The remedy to this unfortunate circumstance is to produce chelates of $Eu^{3+}$ with a ligand that absorbs well and that then transfers the excitation energy to the europium ion via a process very much reminiscent of the Forster intermolecular energy transfer process[16]. The earliest attempt to adapt this scheme to the fingerprint context involved complexing Ruhemann’s purple (RP) with europium and terbium, in much the same way as in the ninhydrin/zinc chloride fingerprint treatment[17]. It should be noted, though, that the manner in which the Zn/RP and Eu/RP complexes operate are very different. In the Zn/RP complex, the zinc ion is, spectroscopically, a spectator ion whose function it is to force the two aromatic moieties of RP into a coplanar configuration, thus rendering the RP, which now becomes orange in color, fluorescent. In the analogous Eu/RP complex, it is the function of the RP only to absorb and transfer energy. The emission of light is from the Eu or Tb ion of the complex. The gain in luminescence of the Eu/RP complex vs. the unreacted Eu salt was found to be small, however. The same applies to the terbium case. The reason here is that the completion of the ninefold coordination of the Eu ion involves waters of hydration. It is well known that such hydration quenches the Eu luminescence. The RP in the complex is intended to serve a dual function. It is the resulting compound from the selective labeling of amino acids in the fingerprint residue, and it also is to function as the sensitizer, via energy transfer, of the Eu luminescence. Attempts to remedy the water quenching of the Eu luminescence have not been successful because the Eu/RP complex is not robust. Aggressive ligands that displace hydration waters also displace the RP. Accordingly, other labeling/sensitizing strategies have been examined, in which one would prepare mixed ligand complexes. One ligand would serve the labeling function and the other the

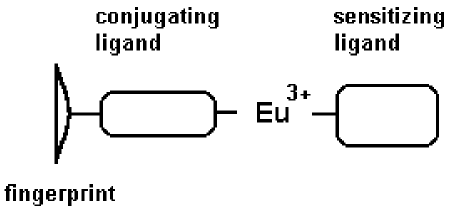

**FIGURE 8.**

Eu luminescence sensitization function, as depicted in Fig. 8. Because in dusting and staining one takes advantage of physical processes of preferential adherence to fingerprint residue, the conjugating ligand in Fig. 8 is actually not needed, but it is in chemical development appropriate to porous surfaces. In 1993, Misner et al.[18] reported on a europium complex with thenoyl trifluoroacetone (TTFA). Shortly thereafter, Lock et al.[19] reported on a mixed-ligand complex involving o-phenanthroline and TTFA. These complexes are effective for dusting and staining purposes. In the past, most chemical fingerprint development strategies have focused on amino acids of fingerprint residue, with less attention paid to lipids in the residue. Such lipid specificity has come into focus since[10] in connection with europium complexes, but until now, fingerprints older than about one week have defied processing. The current effort returns to the labeling of amino acids in one strategy and the labeling in fingerprint residue of either fatty acids or amino acids in another strategy. These labeling strategies are taken up next to set the stage for current R and D work. Much can be gleaned here from the biochemistry literature.

## Fingerprint Labeling Strategies

A carboxylic acid may react with an amine to form an amide, as shown in Fig. 9 (**I**). The reaction is not facile, though, because the OH group of the acid is not a good leaving group. The reaction is therefore usually mediated by a carbodiimide. This mediated amidation reaction is well known in the biochemistry community and is frequently utilized in the labeling of proteins, for instance. The situation in the fingerprint context is somewhat different in that one must have an inherent incompatibility between the labeling reagent and the target material of the fingerprint. That target material must not be soluble in the solvent that delivers the labeling reagent, otherwise the

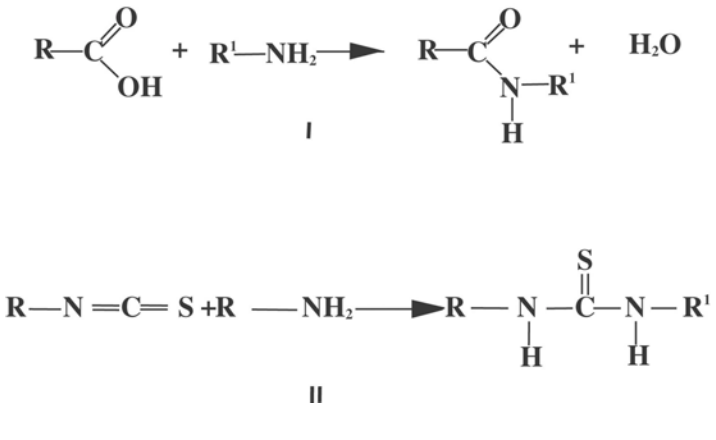

**FIGURE 9.**

fingerprint detail will bleed out to render the fingerprint useless. In other words, in the fingerprint scenario one must have a solid/liquid interfacial reaction that does not significantly affect the structure of the solid. In connection with reaction **I** of Fig. 9, it is to be noted that the carboxylic acid may be the labeling reagent and the amine a constituent of the fingerprint, or the amine may be the labeling agent and the acid a constituent of the fingerprint. The labeling strategy is thus in principle quite flexible.

An isothiocyanate-functionalized labeling compound may react with an amine of the fingerprint residue as shown in Fig. 9 (**II**). This too is a well-known reaction in biochemistry for protein labeling. It is a fairly facile reaction and thus of promise for fingerprint work.

## Lanthanide-based Fingerprint Development Revisited[20]

In keeping with the above two biochemistry-inspired chemical fingerprint labeling strategies, two commercially available protein labels are currently under study. The first is Sypro® Rose Plus Dye, from Molecular Probes (www.probes.com). Preliminary work with it in the author's laboratory (www.phys.ttu.edu/cfs) indicates that old fingerprints on paper can be detected with this label. Unfortunately, the manufacturer declines to disclose the nature of this europium complex. The second label under study is Quantum Dye™, produced by Research Organics (www.resorg.com). The structure of this compound is shown in Fig. 10. Following reaction with fingerprint residue, TTFA is applied for completion of the $Eu^{3+}$ coordination and sensitization of the europium luminescence.

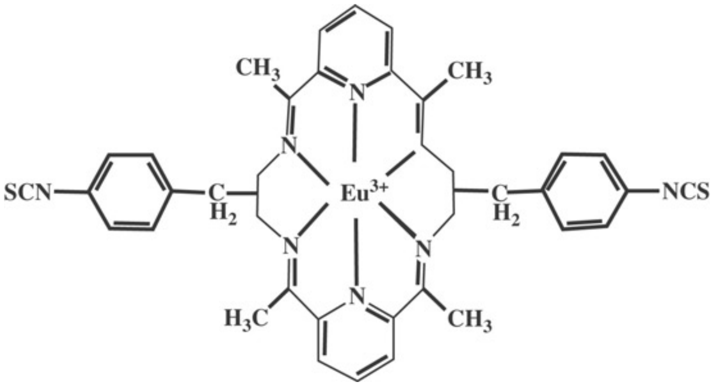

**FIGURE 10.**

## Quantum Dots and Nanoparticles

Semiconductor materials in bulk luminesce rather poorly or not at all. However, when the dimensions of the material are reduced in size to nanometer order, intense luminescence can be obtained. The luminescence typically has several lifetime components, with a substantial fraction of the luminescence of lifetime in the $10^{-8}$- to $10^{-6}$-s range, and is thus suitable to time-resolved imaging schemes. The color and luminescence lifetime can be tailored by adjusting the particle size. The luminescence is relatively sharply defined, much like the luminescence of typical molecules, but the absorption remains broad, much like the band structure absorption of bulk semiconductor material. From this latter perspective, nanocrystals, also referred to as quantum dots, and nanoparticles conveniently lend themselves to a wide range of photoluminescence excitation light sources. The nanoparticles and nanocrystals tend to be robust, not as susceptible as many molecules are to photobleaching. CdS and CdSe are the most commonly employed nanocrystal/nanoparticle materials. CdS particles are usually capped with organic material to prevent aggregation of the nanoparticles and, perhaps, to also achieve functionality for labeling purposes. In the author's laboratory, CdS nanoparticles have been effectively employed for staining latent fingerprints[21]. CdSe nanocrystals are typically capped with zinc sulfide to protect the nanocrystal and also to serve as the site for attachment of conjugating organic ligands designed for labeling purposes[22,23]. Acid and amino-functionalized conjugating ligands are typical. In the author's laboratory, the feasibility of fingerprint detection by staining and chemical development has been demonstrated[24]. Because commercial companies manufacturing these quantum dots have formed only recently (e.g., Quantum Dot Corp., www.qdots.com), CdSe quantum dots have been extremely expensive in the past, being available only in research quantities. Thus, fingerprint work with them has yet to reach maturity. This state of affairs will presumably change shortly as production of such quantum dots, which targets primarily the biomedical community, takes off.

## CdS/dendrimer Nanocomposites[25,26]

If one were to react a solution of sodium sulfide and cadmium nitrate in, e.g., water, cadmium sulfide would form as a bulk precipitate. If, however, the solution contained certain dendrimers, CdS aggregates with the dendrimers would form in which the CdS existed in nanoparticle size[27], displaying the desired luminescence intensities and lifetimes for time-resolved imaging. Again, luminescence color and lifetime can be tailored by adjustment of the solvent system and choice of dendrimer. Suitable dendrimers are the Starburst® (PAMAM) dendrimers available from Aldrich, for instance. They can be obtained with terminal carboxylate and amino functionalities, and thus lend themselves to the usual biolabeling purposes. Both staining and chemical fingerprint development have been demonstrated in the author's laboratory, but the methodology is as yet not sufficiently mature for routine implementation in casework.

## Instrumentation Adaptation

In the described nanoparticle strategies, one deals with lifetimes that are too short for mechanical excitation light chopping as shown in Fig. 4. It is likely that the CW laser and light chopper in this figure will be replaced by a pulsed, frequency-tripled Nd:$YVO_4$ laser. Such lasers have high repetition rates, as needed; have pulse widths of roughly 20 ns, of the right order for nanoparticle luminescence excitation; and operate at about 355 nm, convenient again for nanoparticle luminescence excitation. One might eventually also foresee utilization of LED array lamps and laser diodes. These can be electronically manipulated to turn on and off. At present, their powers are insufficient for fingerprint work, but this may change before long, especially in concert with advances in digital imaging.
